# Electro-Therapeutics

**Published:** 1872-10

**Authors:** A. P. Peck

**Affiliations:** 123 Calumet Avenue; Chicago


ARTICLE II.—*Electro-Therapeutics.* A paper read by request of the "Chicago Society of Physicians and Surgeons," at the regular meeting, July 22nd, 1872. By A. P. PECK, M.D., Chicago.

The principal forms of electrical force used therapeutically are the Galvanic or continuous and the induced or Faradic currents. All forms of static electricity are now little used; although they can be made valuable for medical purposes, they are more generally painful in their application and difficult to manipulate, while nearly all their effects can be produced by the force in a state of lower tension.

The requisites for a medical current are generally that there be a large quantity of electricity of low intensity. Of all kinds of batteries for generating electricity, the one most nearly fulfilling these requisites is the modification of the sulphate of copper battery, known as the specific gravity battery, one excellent form of which is patented by Dr. Hill, of this city, and is in general use on telegraph lines.

The battery is of large bulk, and only suitable for stationary use. The subject of portable batteries is important, but the time does not admit of its discussion here.

Inasmuch as much of the success of the therapeutical application of electricity depends on the kind used, I have deemed it worth while to devote this much space to indicating the best one for the purpose.

THE GALVANIC CURRENT.

This is sometimes called the primary current, the battery current, the continuous current, or the interrupted continuous current. The term I prefer, and the one most generally employed by scientific medical electricians, is the one which heads the list, viz., the galvanic current.

This you are aware is obtained from a large number of elements combined, so that the positive plate of one element is connected with the negative plate of the next, the whole thus forming a consecutive series called a "compound battery," the current being taken from the two ends, one of which will be positive and the other negative.

The action of the current seems to be primarily and powerfully upon the nervous system. It is thus useful in the large class of neuroses, called neuralgias, where our pathological researches show us no discernible anatomical changes. Among these belong the hyperæsthesias—a class of maladies in my opinion not sufficiently estimated.

This current possesses the power of impressing the nerves of special sense, and is therefore very valuable in affections of those nerves requiring a stimulant action to rouse them to their normal activity, as in atrophies and degenerations.

Inasmuch as many of these affections are prone to affect the cranial nerves, I will here state that when this current is used about the head, where it is liable to pass through the brain or an important nerve trunk, like the vagus, at least three pieces of apparatus should be used: a rheotrope or pole-changer, for reversing the direction of the current; a rheometer, for measuring; and a rheostat, for graduating its strength. These should be used both for exercising a prudent care lest injury should result to the brain or viscus supplied by the nerve, and for the more accurate therapeutical application which can be effected by their use. To these may be added a rheotome or current-breaker, for interrupting the current slowly or rapidly as desired—a proceeding often useful both in treatment and diagnosis.

One of the most important effects of this current is its power of relaxing spasm. Next to chloroform and the ethers, it is perhaps the most powerful anti-spasmodic known, even tetanic spasms yielding to its influence, and the clonic cramps of the algid stage of cholera being relieved by its appropriate application.

So, too, that painful affection known as hemicrania, which oftentimes seems to be a true tetanus of the vaso-motor nerves, may be relieved by galvanization of the sympathetic.

We may take advantage of the chemical action of this current to produce electrolytic effects on the morphological and heterologous constituents of the body: collections of fluid in serous sacks being most amenable to this treatment, and ranging with varying degrees of efficiency through numerous conditions of degenerate cell action; even the electrolysis of cancerous tumors being advised by high authority when operative interference is deemed unadvisable.

Because of the intimate correlation of this force to the heat force we are able, by its retardation in a loop of the poorly conducting metal platinum, to produce a sufficient degree of heat for the actual cautery.

With regard to its medical use it is unfortunate that we have no means of correctly indicating the strength of the current used. The expression, "a current from so many cells of a certain kind of battery," is too vague, as all batteries vary in strength from time to time, and there is no accurate standard of comparison between any two. And I confess I do not know at present of any way to express, in a satisfactory manner, the strength of a galvanic current used, but hope that a standard rheometer will be sometime introduced, that there may be accuracy and uniformity in all applications.

THE INDUCED CURRENT.

The battery used for obtaining this current should consist of one large cell, and furnish a sufficient quantity of electricity.

From nearly all medical induction coils two currents are obtained, one called the primary or extra current, and the other the secondary. The first is taken by branch wires from the first or inducing coil of the helix, and is merely the battery current broken by the rheotome, and intensified by the inductive action of the coils on each other. The other is the induced current proper, and is taken from the outer coil which has no connection with the battery. And inasmuch as the duration of an induced current is only momentary, namely, upon the making and breaking of the inducing current, a rheotome or current-breaker is always introduced into the primary circuit, so that the current as felt is always a series of shocks. With the quantity current battery, however, and a properly constructed helix, these shocks are not painful. You will thus recognize the difference between the current as it should be and the one obtained from most of the small portable batteries, put up and sold for medical use, invariably as the best in the market, but which are so objectionable from their small quantity and fierce biting intensity of their currents, that they should be banished from medical use, except in a small minority of cases.

The instruments used for applying electricity are called rheophores, and many different forms of these are described in books and put up by enterprising instrument makers. But, however imposing and scientific their appearance, or astonishing their cost, this vast array of complicated mechanism is unnecessary for ordinary use, and a few simple forms are enough.

As we said of the galvanic current, that it acted primarily and powerfully upon the nervous system, so we may say of the induced, that whether we use the primary or secondary current, its most noticeable effect is upon the muscular system. This is owing to the fact that every time a current of electricity is passed through a muscle it causes it to contract, and as the faradic is a constant succession of currents there is a succession of contractions, which, if sufficiently rapidly produced, may amount to spasm. The ordinary method of applying this current is by means of sponges to various parts of the body; and it is a very efficient way, but far exceeded in power by the electro-thermal bath—a method of use which you will not find described in books, nor has it been used anywhere as long and successfully as in Chicago.

It is a bath-tub of non-conducting material, with the rheophores arranged along the sides, so that the electricity can be sent in any direction through the water, including in its action the patient who is placed therein.

The advantages of this method are many, among which are these: The patient need not be touched by the hands of the operator, the direction of the currents being perfectly governed by means of a key-board. The avoidance of concentrating the current on any one part, as in the application with sponges, and the consequent avoidance of shock to any part, as the spine. The certainty of the application is not lessened, while if a local treatment of any part is desired at the same time, it can be made through a sponge to the part radiating the electricity from that point on all sides.

Bearing in mind the effect of this form of the force on muscle cell, whether of the striped or unstriped variety, it will be readily seen how there is not so efficient a treatment as this for obstinate constipation in all the range of therapeutics. The contractions of muscle produced by electricity are perfectly physiological, and the renewal of tissue thus obtained is permanent and normal; and all the drugs of the pharmacopœia, pummelings of the movement cure, change of climate, or of diet, could not do it as well. If the curing of constipation were the only thing we could do with it, would it not be deserving of high praise? But all degenerated muscles are acted on in the same way, and if enough of the contractile fibre cells are left, the nutrition may be so improved that it shall be restored to its normal condition.

This property of acting on contractile fibres enables us to control the formation of hemorrhoids, to collapse vascular tumors, promote uterine contractions, and restore the tonicity of a dilated bladder.

In glaucoma the application of this current often renders the operation of iridectomy unnecessary, by producing absorption of the effused fluid.

But besides these dynamic effects the application of induced electricity has other purposes. It also acts on the nervous system, but in a more general way than galvanism.

We often meet cases in which there is malassimilation of food, and although the patient eats enough he is literally starving in the midst of plenty. There the application of faradism through the medium of the bath has the happiest effect, and rouses to duty the dormant powers through whose dereliction the tide of life is turned aside. In the control of pain this current rivals galvanism; and, contradictory as it may seem, sometimes relaxes spasm better.

In short, I may as well admit here that all we know of the physiological action of the two currents, does not enable us to decide arbitrarily which will benefit every case. Very often both are needed. Is there not then need of the most careful study and intelligent observation, that a rational system of therapeutics may be established in relation to this agent, so powerful for good or harm? But the time fails me to enumerate the vast number of points in which electricity has a bearing on the animal economy.

I cannot speak of its wonderful control over rheumatism, nor the speed with which a sprained ligament will get well under its influence, nor its control over certain inflammations, nor the power it has in promoting the absorption of many tumors and indurations, though all of these are fruitful topics, and many instances in point of each could be adduced from cases in actual practice. My aim has been in this paper to group together a part of the many therapeutical uses of electricity. I have left much unsaid, and yet have made many points you cannot find in the books.

It is a notorious fact that in no department of medicine are the books up to the latest improvements, and especially is this true of the rapidly growing science of electro-therapeutics.

In conclusion, let me urge you to a habit of close observation, when you use this agent, and when reporting upon it, a clear understanding of terms and a correct nomenclature. For I would protest against the careless manner in which it is used, and judgment formed of it. Where in all the range of scientific research would men be allowed to base opinions upon such practice as to place two ridiculous tin handles in the hands of a patient, send a rasping current of electricity through him, and then proclaim, "It's no use, I've tried electricity and it don't amount to anything," etc., *ad nauseam?* It would be called brazen empiricism and quackery, as indeed it is.

123 Calumet Avenue.

